# Comment on “Effect of Multilaminate Small Intestinal Submucosa as a Barrier Membrane on Bone Formation in a Rabbit Mandible Defect Model”

**DOI:** 10.1155/2020/7624154

**Published:** 2020-08-14

**Authors:** Nazanin Daei, Siavash Ahmadi-Noorbakhsh

**Affiliations:** ^1^Department of Clinical Pathology, Faculty of Veterinary Medicine, University of Tehran, Tehran, Iran; ^2^School of Human Sciences, The University of Western Australia, 35 Stirling Highway, 6009 Crawley, Australia

We read with interest the article by Wu et al. entitled “Effect of Multilaminate Small Intestinal Submucosa as a Barrier Membrane on Bone Formation in a Rabbit Mandible Defect Model” which was published in the journal of *BioMed Research International* [1]. The authors showed a great potential for multilaminate small intestinal submucosa to be used as a barrier membrane for guiding bone regeneration. They used *in vitro* and *in vivo* models to investigate the proposed treatment. For the *in vivo* model, 25 laboratory rabbits underwent soft tissue (nine rabbits with eight subcutaneous punches on the back of each) or orthopedic surgeries (two 8 mm width mandibular defects on the remaining rabbits). While the main objective of the research was achieved successfully, there were some technical and ethical issues regarding the method of anesthesia used in this research. As veterinary surgeons, we would like to use this opportunity to have an input in this regard.

The authors stated using “2% sodium pentobarbital administered at 30 mg/kg via intravenous injection” for creating the state of “general anesthesia” for performing soft tissue and orthopedic procedures on animals [1]. Although sodium pentobarbital (pentobarbitone) may induce a sleep-like state and mild anesthesia, it cannot provide analgesia required for a painless surgery.

In this regard, we would need to differentiate between “general anesthesia” and “surgical general anesthesia”. General anesthesia (GA) refers to loss of consciousness due to the use of drugs that create a state of reversible CNS depression [2]. Patients under GA are “not arousable by noxious stimulation” [2] but it does not necessarily mean that they cannot sense or perceive pain. In fact, pain sensation (nociception) is not affected by consciousness, and as long as the chain of transduction, transmission, and modulation of noxious stimuli is intact, nociception continues even during GA [2]. Nociception further leads to “conscious perception of pain” (or nociperception) [2]. Therefore, surgical general anesthesia (SGA), which is required for painless surgery, can be defined as GA that consists of four parts: loss of consciousness, pain control, muscular relaxation, and amnesia [2].

Pentobarbitone is a short-acting oxybarbiturate [3] and does not provide antinociception or analgesia required for soft tissue or orthopedic surgeries in rabbits [2, 3]. This drug has a narrow therapeutic window and may cause profound cardiovascular and respiratory depression in doses very close to its therapeutic dose. Pentobarbitone can only induce a light to medium GA following IV administration at the dose range of 30–45 mg/kg, and higher doses might cause respiratory arrest before SGA can be achieved [3]. Therefore, it is recommended to avoid using pentobarbitone for survival surgeries in rabbits, and the use of this drug is mainly limited to some nonsurvival surgeries or as a main ingredient in many chemical euthanasia agents [3]. When using pentobarbitone as the sole anesthetic agent, the level of CNS depression may be as high as to prevent voluntary movement of the animals in response to pain. However, this can never guarantee a painless surgery, especially during the anesthetic recovery, when the CNS concentration of the drug (and thus the CNS depression) is declining.

For a proper short-term (30 min) SGA, one may consider providing preanesthetic medications consisting of at least a sedative/tranquilizer and analgesics. In rabbits, this may be achieved by an IM injection of Hypnorm (i.e., 0.315 mg/ml fentanyl and 10 mg/ml fluanisone) at a dose rate of 0.2–0.5 ml/kg [3] accompanied by an NSAID (such as meloxicam or carprofen), if possible. Ten to fifteen minutes following the administration of Hypnorm, an IV line can be established, and a slow IV injection of midazolam or diazepam (both at 0.5 mg/kg) can be used to provide unconsciousness and muscular relaxation [3], thus meeting all four components of SGA. For multimodal analgesia in the study under discussion, one may consider a splash block of the skull's periosteum, using a drop of 2% lidocaine. Additional IM injections of Hypnorm (0.1 ml/kg Hypnorm diluted 1 : 10 with water for injection and NOT saline) every 30-40 minutes can be used to prolong the anesthesia [3] for less than two hours. For longer anesthetic periods, one can consider other anesthetic protocols such as administrating IV fentanyl (30–100 *μ*g/kg/h) instead of additional doses of Hypnorm [3]. If using IV fentanyl, it is required to maintain a secure airway access to ventilate the animals if fentanyl-related respiratory depression occurs. The anesthetic recovery can be expedited using slow IV injection of nalbuphine (0.1 mg/kg) or buprenorphine (0.01 mg/kg) [3], titrated to effect.

Postoperative pain relief should be tailored according to the type of pain (such as visceral vs. somatic or chemical vs. mechanical), severity and duration of pain, and the effect of analgesics on research data. At best, multimodal analgesia can be achieved by using a combination of drugs such as NSAIDs (e.g., PO or SC meloxicam 0.1-0.3 mg/kg every 24 h or Carprofen 1.5-5.0 mg/kg every 12-24 h, considering the probable renal and gastrointestinal side effects of these drugs) and/or opioids (SC or IM buprenorphine 0.01-0.05 mg/kg every 6-12 h), and/or local anesthetics [4]. Good practice is to administer the analgesics before the onset of pain perception (i.e., preemptive analgesia). Any withholding of analgesics or proper anesthetic method should be justified by strong scientific reasons, and the reason must be ethically justifiable.

The aim of this letter was not to review various rabbit anesthesia techniques, and a dozen other established anesthetic/analgesic protocols can be considered to fit the requirement of a particular study. Laboratory animals are the very fragile contact point between our research ideas and real-world data. With proper care of the animals, more accurate research data can be achieved, and most importantly, the welfare of these living beings can be preserved.

## References

[1] Wu W., Li B., Li Y., Wang X., Tang L. (2018). Effect of Multilaminate Small Intestinal Submucosa as a Barrier Membrane on Bone Formation in a Rabbit Mandible Defect Model. *BioMed Research International*.

[2] Grimm K. A., Lamont L. A., Tranquilli W. J., Greene S. A., Robertson S. A. (2015). *Veterinary Anesthesia and Analgesia: The Fifth Edition of Lumb and Jones*.

[3] Flecknell P. (2015). *Laboratory Animal Anaesthesia*.

[4] Quesenberry K., Carpenter J. (2011). *Ferrets, Rabbits, and Rodents*.

